# Application of transcranial alternating current stimulation to improve eSports-related cognitive performance

**DOI:** 10.3389/fnins.2024.1308370

**Published:** 2024-02-27

**Authors:** Fujia Jiao, Jie Zhuang, Michael A. Nitsche, Zhenggen Lin, Yuanbo Ma, Yu Liu

**Affiliations:** ^1^Key Laboratory of Exercise and Health Sciences of Ministry of Education, Shanghai University of Sport, Shanghai, China; ^2^Department Psychology and Neurosciences, Leibniz Research Centre for Working Environment and Human Factors, Dortmund, Germany; ^3^School of Psychology, Shanghai University of Sport, Shanghai, China; ^4^University Hospital OWL, Protestant Hospital of Bethel Foundation, University Clinic of Psychiatry and Psychotherapy and University Clinic of Child and Adolescent Psychiatry and Psychotherapy, Bielefeld University, Bielefeld, Germany; ^5^German Center for Mental Health (DZPG), Bochum, Germany; ^6^Department of Psychology, Ruhr University Bochum, Bochum, Germany

**Keywords:** eSports, visual attention, transcranial alternating current stimulation, visual spatial attention distraction task, eSports skill performance

## Abstract

**Introduction:**

Electronic Sports (eSports) is a popular and still emerging sport. Multiplayer Online Battle Arena (MOBA) and First/Third Person Shooting Games (FPS/TPS) require excellent visual attention abilities. Visual attention involves specific frontal and parietal areas, and is associated with alpha coherence. Transcranial alternating current stimulation (tACS) is a principally suitable tool to improve cognitive functions by modulation of regional oscillatory cortical networks that alters regional and larger network connectivity.

**Methods:**

In this single-blinded crossover study, 27 healthy college students were recruited and exposed to 10 Hz tACS of the right frontoparietal network. Subjects conducted a Visual Spatial Attention Distraction task in three phases: T0 (pre-stimulation), T1 (during stimulation), T2 (after-stimulation), and an eSports performance task which contained three games (“Exact Aiming,” “Flick Aiming,” “Press Reaction”) before and after stimulation.

**Results:**

The results showed performance improvements in the “Exact Aiming” task and hint for a prevention of reaction time performance decline in the “Press Reaction” task in the real, as compared to the sham stimulation group. We also found a significant decrease of reaction time in the visual spatial attention distraction task at T1 compared to T0 in the real, but not sham intervention group. However, accuracy and inverse efficiency scores (IES) did not differ between intervention groups in this task.

**Discussion:**

These results suggest that 10 Hz tACS over the right frontal and parietal cortex might improve eSports-related skill performance in specific tasks, and also improve visual attention in healthy students during stimulation. This tACS protocol is a potential tool to modulate neurocognitive performance involving tracking targets, and might be a foundation for the development of a new concept to enhance eSports performance. This will require however proof in real life scenarios, as well optimization.

## 1 Introduction

Electronic Sports (eSports) is an entertainment activity with increasing popularity in the 21st century, attractive for both, participants and spectators, and gains popularity mainly in young people (Chung et al., 2019; Nagorsky and Wiemeyer, 2020). eSports games require fast-paced, constant attention to movement, quick decision-making, good hand-eye coordination, and excellent combat and reaction time performance. eSports include especially Action Video Games (AVG), such as Multiplayer Online Battle Arena (MOBA), and First/Third Person Shooting Games (FPS/TPS) (Bediou et al., 2018). In contrast to traditional sports, where the success of athletes relies critically on the development and performance of complex motor skills, eSports athletes depend more on the development of cognitive functions, such as visual attention, decision-making skills, and reaction time (Himmelstein et al., 2017). Many studies show that AVG players have better visual attention than non-gamers, and that non-gamers can improve their cognitive abilities to some extent by game training (Green and Bavelier, 2003, 2006a,b).

Most of the studies conducted so far are cross-sectional studies to compare visual attention of AVG players and non-video game players. As early as 2003, it was reported that AVG players have better visual attention than non-players and that training of non-players improved visual attention (Green and Bavelier, 2003). In subsequent studies, it was reported that playing video games improved performance of various attentional and perceptual tasks, and similarly, performance of a functional visual field task was improved by video game experience (Green and Bavelier, 2010, 2015). Moreover, He et al. reported that AVG players had better attentional control in visual computer tasks, but speculated that transfer of performance gains to other tasks might be limited (He et al., 2022). Prolonged gaming is demanding with respect to attention control and related cognitive functions (Bavelier and Green, 2019). AVG games are considered to require cognitive skill training, including task switching, visuomotor coordination, processing speed, and attentional control in addition to enhancement of general physical fitness (Feng et al., 2007; Boot et al., 2008; Green and Bavelier, 2015; Billieux et al., 2017). However, regarding cognitive training, besides the daily gaming routine not much research focuses on improvement of cognitive functions of eSports athletes, including focused improvement of visual attention (Green and Bavelier, 2006a; Bediou et al., 2018).

Non-invasive Brain Stimulation (NIBS) is a potential tool for enhancing neurocognition in eSports athletes (Zhuang et al., 2020; Machado et al., 2021). NIBS includes a group of neuromodulation techniques that has been developed over the last three decades to modulate brain activity, as well as relevant cognitive and motor functions. Transcranial Magnetic Stimulation (TMS), based on electromagnetic principles, and Transcranial Electrical Stimulation (tES), based on the application of a weak, painless current to the scalp, are the most common methods used in NIBS (Polanía et al., 2018). In transcranial direct current stimulation (tDCS), a weak direct current is administered via electrodes placed on the head that depolarizes or hyperpolarizes the resting membrane potential of neurons, dependent on the direction of current flow, thereby altering cortical excitability (Nitsche and Paulus, 2000). Transcranial alternating current stimulation (tACS) uses oscillatory electrical stimulation to promote neuronal activity in specific frequency bands (Cabral-Calderin and Wilke, 2020; Riddle and Frohlich, 2021). Recent studies have moreover shown that tACS improves cognitive functions by enhancing brain oscillations that synchronize activity between distant brain regions (Herrmann et al., 2013; Johannes et al., 2018; Cabral-Calderin and Wilke, 2020). Indeed, studies have shown that stimulation of dual brain regions can improve synchronization of remote brain regions and thus improve cognitive function (Reinhart and Nguyen, 2019; Hosseinian et al., 2021).

Resting-state functional imaging data showed that blood oxygen level-dependent (BOLD) activity in the frontoparietal network correlates with alpha power (Sadaghiani et al., 2012). Zanto et al. (2010, 2011) observed increased alpha phase coherence between the right prefrontal and visual cortices during attention task performance. Visuospatial attention is associated with robust and sustained long-range synchronization of cortical oscillations exclusively in the high-alpha (10–14 Hz) frequency band. This synchronization between frontal, parietal and visual regions was observed concurrently with amplitude suppression of low-alpha (6–9 Hz) band oscillations in the visual cortex (Lobier et al., 2018). van Schouwenburg et al. (2016) found preliminary evidence that phase coherence enhancements via tACS of the right frontoparietal network may play a crucial role for top-down control of spatial attention, since enhanced alpha synchrony in the right frontal and parietal cortices was associated with improved performance in a visuospatial attention task. Therefore, alpha tACS might be suited to improve eSports performance related to visual attention involving the right frontoparietal network.

Although neuromodulation via non-invasive brain stimulation has been proposed as a performance-enhancing tool in eSports, still only few respective studies are available, and only one study reported an improving effect of tDCS on digital game performance (Zhuang et al., 2020; Friehs et al., 2021). In the current study, based on knowledge about the involvement of alpha activity and synchronization of the right hemispheric frontoparietal network, we hypothesized that strengthening of respective alpha activity via tACS will improve visual attention performance, and that for this improvement synchronization of frontal and parietal network components is required in eSports players. Hereby, we intended to probe whether tACS has potential as an effective tool to improve gaming performance via enhancement of visuospatial attention, and expected that main components of gaming performance, such as accuracy, quickness, and reflex-like reactions would improve by this intervention.

## 2 Materials and method

### 2.1 Participants

We recruited 27 healthy students (20 female, 7 male) from Shanghai Sport University, who had never played MOBA or FPS genre games (PC games or mobile games were included), and did not play AVG within 6 months before the experiments. Exclusion criteria were (1) Alcohol abuse and smoking, (2) presence of personal or family neurological disease, metal implants or electronic devices in the head, or a history of head injury, (3) presence of cardiovascular disease (e.g., high blood pressure), (4) serious medical conditions, history of upper limb injury within the last 6 months, (5) history of brain surgery, and metal in the head, (6) cognitive impairment, psychiatric disease, and (7) long-term or recent use of CNS-active medication. All subjects gave written informed consent before participation, and the study was approved by the ethical committee of Shanghai University of Sport (approval number: 102772022RT047).

### 2.2 Procedure

**FIGURE 1:**
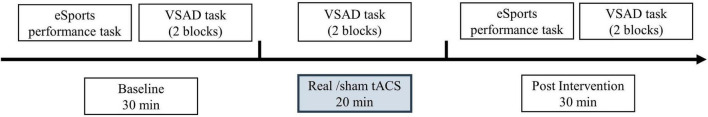
The course of the experimental sessions depicted in the top lines show the tasks the volunteers performed at respective time points, the bottom line indexes the duration of respective activities, and the middle line is spaced by black rectangles, subdividing baseline, intervention, and post-intervention time slots. The interval between the two sessions was 1 week minimum. VSAD task, visual spatial attention distraction task; tACS, transcranial alternating current stimulation; eSports performance task, this task contained three fixed eSports ability test games.

This study was conducted in a crossover within-subject design. Before the experiment, participants were first conducting an eSports performance task, which was divided into three parts, each of which included a practice part. We chose a test of three games as the eSports performance task, and the duration of this task was about 10 min. Next, the participants were asked to conduct the visual spatial attention distraction task, which includes components of selective visuo-spatial, object-based and feature-based attention (for details refer to section “2.3 Visual spatial attention distraction task”) (Maunsell and Treue, 2006; Cohen and Maunsell, 2011). The presentation order of 4 different blocks with different trial order in each experimental session was determined by two pseudorandomized orders A, and B (A: pre-12, middle-13, after-34; B: pre-12, middle- 24, after- 34). The association between order, and intervention condition was randomized between participants. Before the formal test, a practice session of about 24 trails was conducted to ensure an accuracy rate of above 70% in that test. Then the formal visual spatial attention distraction task which contained 2 blocks (one block including 152 trials) was performed. Task duration was about 20 min. Next, subjects were exposed to 20 min tACS (sham or real), and conducted the visual spatial attention distraction task again simultaneously. After tACS, the participants first performed the eSports task and then continued with the visual spatial attention distraction task. The total course of an experimental session is shown in Figure 1. Side effects and blinding success were tested at the end of the experiment via questionnaires (Zhang et al., 2022). The day before the experiment, participants were asked not to stay up late, not to drink coffee, alcohol or consume other central nervous system-affecting drinks or drugs within 24 h before the test, and not to perform strenuous physical activity within 24 h before the experiment. An interval of at least 1 week between sessions was obligatory.

### 2.3 Visual spatial attention distraction task

**FIGURE 2:**
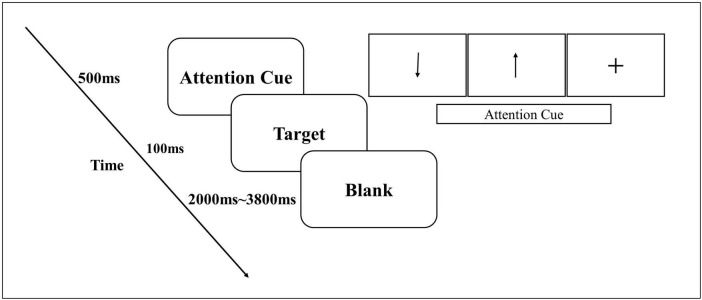
Schematic overview of the Visual Spatial Attention Distraction Task. The attention cue induced a bias (arrow), or no bias (+), the flow chart on the left shows the duration of stimulus presentation, and the time it took for completion of a trial. The duration of the task including two blocks was about 20 min.

The experimental stimuli were displayed using E-Prime, version 3.0 (Psychology Software Tools Inc., USA). Each trial began with an attentional cue presented for 500 ms, followed by a target for 100 ms, and a blank screen for 2000∼3800 ms. Participants were instructed to respond as quickly as possible by pressing a button of the mouse when the target picture appeared. During the experiment, participants were asked to identify whether a blue butterfly was shown on the left or right side of the picture, by pressing the left or right mouse button with the index or middle finger of the right hand, respectively. Before the start of the main experiment, the participants performed a short practice session of 24 trials to get familiar with the experimental procedure. Participants performed two blocks before, 2 blocks during, and 2 blocks after intervention, each block was composed of 152 trials. The duration of 2 blocks was about 20 min (Figure 2).

**FIGURE 3:**
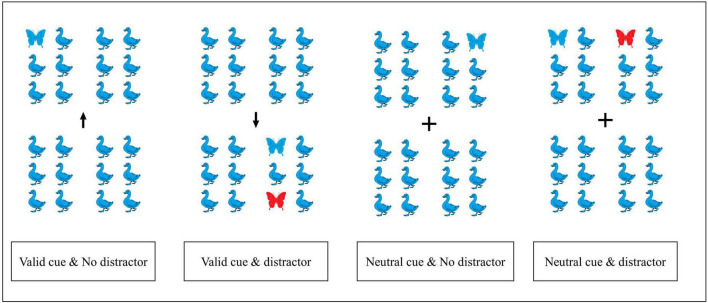
Shown are the four target categories. Participants were asked to identify if the blue butterfly was positioned on the **left** or **right** side. The red butterfly served as distractor. The arrow represents the valid cue, and its direction, “+” represents the neutral cue.

The experimental design included two factors, namely distraction (with or without a distractor) and divided attention (valid or neutral cue) (Figure 3). An arrow symbol in the middle of each picture served as valid attentional cue, and directed attention to either the upper or lower panel of the picture, and the target (blue butterfly) always appeared in the panel indicated by the valid cue. In contrast, the “+” symbol, which served as alternative attentional cue, did not cue attention to either panel, here the target appeared with identical probability in either panel. The target pictures were divided into 4 categories: valid cue with distraction (52 experimental trials), valid cue without distraction (24 trials), neutral cue with distraction (52 trials), and neutral cue without distraction (24 trials) (Figure 3).

### 2.4 eSports performance task

The eSports performance task was performed on an ASUS desktop computer (CPU: Intel Core i7-10700k, GPU: NVIDIA Quadro P2200, RAM: Corsair DDR4 3600Mhz) connected to a standard full HD monitor, mouse and keyboard. The complete test procedure was explained to the participants before starting the test, and a practice test was conducted before the start of the specific test before intervention. The sensitivity of the mouse was adjusted to the comfort level of each participant.

We used the following three tests: “Exact Aiming,” “Flick Aiming,” and “Press Reaction” (Aimtastic video game).^1^ In these three tests, the default difficulty level was used. In “Exact Aiming,” bubbles of different size appeared on the screen for 100 s in total, which changed from small to large and then to small again, and had to be tapped before they disappeared. Targets appeared at higher frequency with increasing task duration. First, two bubbles appeared simultaneously in three trials (total duration of these trials 15 s), then the number of bubbles was increased by one up to eight, and each step was repeated 3 times with the exception of the 8 bubbles condition, which appeared only in two trials (total duration of these latter trials 10 s). The total number of trials was 124. Test scores were calculated based on the relation of hits and misses (100 points for hitting the target, minus 50 points for missing one). In the “Flick Aiming” test, first a red ball appears in the middle of the screen. Afterward, a white bubble randomly appears. The participants need to click on the white bubble, and then move the mouse over the red bubble to make it turn green. The duration of the “Flick Aiming” task was approximately 100 s. The test result was scored with 100 points added for a hit and 10 points subtracted for a miss, and 100 points added for a very accurate mouse path (in terms of the percentage value of a straight path of the mouse movement). In the “Press Reaction” test, only one target appears in the center of the screen at a random time point. The participants were instructed to point and click on the target as quickly as possible. The total trial number in the “Press Reaction” test was 10, outcome measures were reaction time for each trial, and an average reaction time was calculated over all trials. Conduction of all of these tasks took 10 min. For all performance tests, participants conducted a practice test before the start of the main experiment. and performed the tests before and after stimulation in the main experiment.

### 2.5 Transcranial alternating current stimulation

Stimulation was performed via a StarStim device (Neuroelectric, Barcelona, Spain) using round Ag/AgCl electrodes (Pistim) with a contact area of 3.14 cm^2^. Electrode impedance was kept below 10 kΩ. Our stimulation protocol was designed to increase alpha coherence between the right frontal and parietal cortices.

**FIGURE 4:**
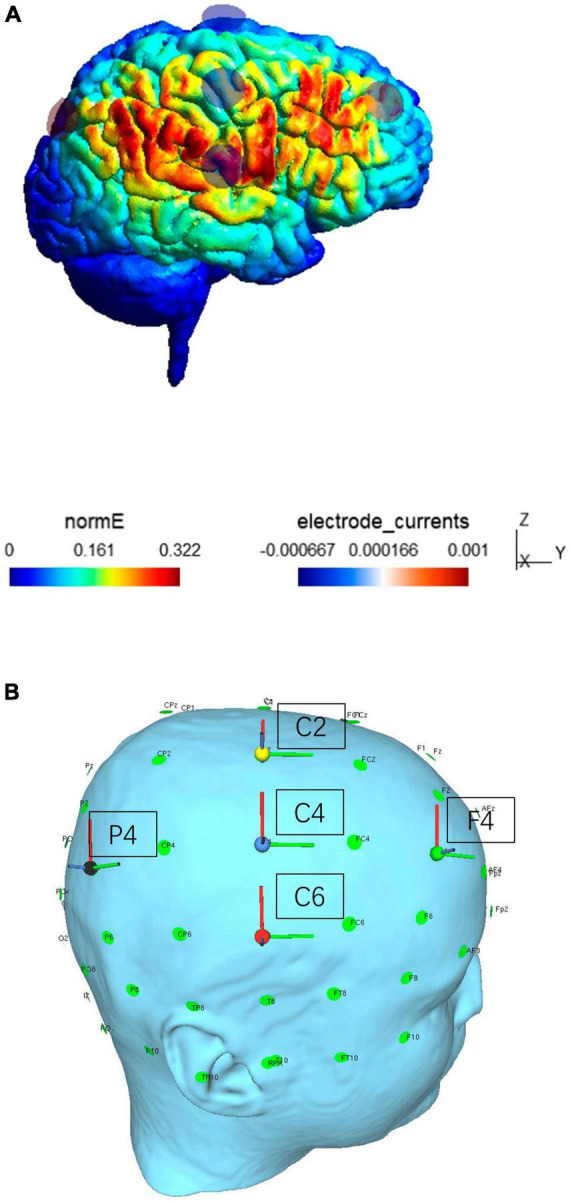
**(A)** Simulation of the electric field induced by tACS in this study. Colors indicate electrical field strength (nE, V/m), from low (blue) to large (red). **(B)** Shows the position of the stimulation electrodes, which were placed over F4 and P4, and the return electrodes, which were placed over C2, C4, and C6 positions.

In this experiment, based on previous research (van Schouwenburg et al., 2016), to stimulate the right frontoparietal network we placed the frontal stimulation electrode over F4. In addition, we placed the parietal stimulation electrode at P4, in the center of the posterior parietal cortex. The frontal and parietal cortices are integral parts of the fronto-parietal dorsal attention network, which is crucially involved in spatial attention (Harris et al., 2008; Martinaud et al., 2016), object-based attention (Hoba et al., 2022) and feature-based attention (Lanssens et al., 2020, 2022), which are all presumed to be relevant for gaming performance. Frequency was set to: α = 10 Hz, the stimulation lasted for 20 min, and in-phase stimulation with the electrodes over F4 and P4 with a sinusoidal alternating current with a peak to baseline intensity of 1000 μA was conducted. The return electrodes were placed at C2, C4, and C6 in order to disperse the heterophasic current over a larger area of disinterest. For stimulation blocks, in sham stimulation, current was immediately ramped up for 15 s and ramped down for 15 s, and again ramped up and down for 30 s at the end, and total stimulation duration was 20 min. The real stimulation was ramped up for 30 s at the beginning, then constant for 19 min, and then ramped down for 30 s. The electrical fields induced by tACS were calculated via the transcranial electrical stimulation simulation software SimNIBS (SimNIBS, University of Denmark, Denmark), and results are shown in Figure 4.

### 2.6 Behavioral analysis

First, only for the VSAD task, we conducted three four-way ANOVAs with stimulation (real vs. sham), phase (pre vs. within vs. post-intervention), attention (valid vs. neutral cue) and conflict (distractor vs. no distractor) as within-subjects factors, and accuracy, reaction time (RT) and inverse efficiency scores (IES) (Liesefeld and Janczyk, 2019) as dependent variables. Accuracy (number of correct trials divided by the total number of trials), and the individual means of RT of the correct trials were calculated. Next, to account for a potential speed-accuracy trade-off, we calculated and analyzed the inverse efficiency score (IES) of each subject and condition, which was defined as mean reaction time of correct trials divided by accuracy. Two-way repeated measures ANOVAs were calculated for the eSports performance tasks. Stimulation (real vs. sham) and Time (pre vs. post) served as within-subject factors, and “Exact Aiming” scores, “Flick Aiming” scores and “Press Reaction” scores as dependent variables. Mauchly’s tests were used to test the assumption of sphericity. In case of a violation of this assumption, the Greenhouse–Geisser correction was applied. In case of significant results of the ANOVAs, Bonferroni’s-corrected *post-hoc t*-tests tests were conducted. The level of significance was set to *P* ≤ 0.05 for all tests.

To examine side effects (e.g., pain, itchiness, and burning) induced by stimulation, Mann-Whitney *U*-tests were used to compare ordinal-scaled variables described as numerical outcomes, based on information from the side-effect questionnaire. To verify blinding success, we included generalized estimating equations of the guessed stimulation conditions (“real tACS,” “sham tACS,” “I do not know”), and the actual stimulation condition (real tACS, sham) as factors. A Chi-square test was applied to evaluate blinding success.

## 3 Results

### 3.1 Side effects and blinding

The majority of subjects stated none or mild side effects. Mann-Whitney *U*-tests showed no significant difference in the ratings of side effects between the two groups (all *p* = 0.21–0.65). The majority of side effect ratings were “none,” and “mild” (Table 1). The Chi-square test showed that blinding was successful (*p* = 0.884) (Table 2).

**TABLE 1:** Side effect ratings in two groups.

Stimulation	Real				Sham			
	None	Mild	Moderate	Severe	None	Mild	Moderate	Severe
Tingling	9	16	2	0	14	12	1	0
Itching	23	3	1	0	21	6	0	0
Burning	26	1	0	0	23	3	1	0
Pain	23	4	0	0	22	4	1	0
Skin redness	25	2	0	0	27	0	0	0
Fatigue	15	9	3	0	16	9	1	1
Phosphenes	24	3	0	0	23	3	1	0

**TABLE 2:** Blinding effect in two groups.

	Real stimulation	Sham stimulation
Correct guess	12	16
Wrong guess	15	11

### 3.2 Visual spatial distraction task

**FIGURE 5:**
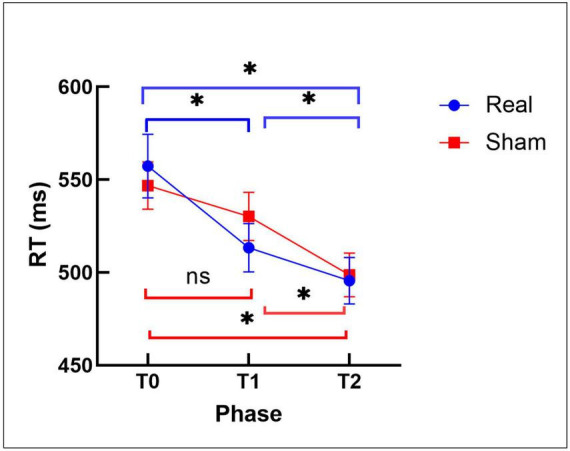
This figure shows VDST task reaction time in different phases (T0, T1, T2) in real and sham stimulation conditions. The figure shows that RT significantly differs between the three phases (T0 > T1, *p* < 0.001; T0 > T2, *p* < 0.001; T1 > T2, *p* = 0.003) in the real stimulation, but only in two phases in the sham stimulation group (T0 > T2, *p* < 0.001; T1 > T2, *p* = 0.009). Error bars show standard error of the mean. Significant results of the *post-hoc t*-tests are marked with asterisks. ns = not significant.

We performed a four-way repeated measure ANOVA on reaction time (RT), including stimulation (real vs. sham), phase (T0, T1, T2), conflict (distractor vs. no distractor) and attention (valid and neutral cue) as factors. The results revealed significant main effects of time [*F*_(2,52)_ = 27.973, *p* < 0.001], conflict [*F*_(1,26)_ = 12.618, *p* = 0.001], and attention [*F*_(1,26)_ = 111.536, *p* < 0.001], but no main effect of stimulation [*F*_(1,26)_ = 0.162, *p* = 0.691]. However, a significant stimulation × phase interaction was found for RT [*F*_(2,54)_ = 4.28, *p* = 0.021]. All other interactions were not significant (Table 3). The *post-hoc* tests showed significant RT effects in the real stimulation group in all phases (T0 > T1, *p* < 0.001; T0 > T2, *p* < 0.001; T1 > T2, *p* = 0.003), but only in two phases in the sham group (T0 > T2, *p* < 0.001; T1 > T2, *p* = 0.009). However, there were no significant differences between real and sham stimulation. Only for T1 a trend wise difference was detected [*P* = 0.098, real (513.33 ± 13.04) < sham (530.201 ± 13.04)] (Figure 5). Additionally, we performed four-way repeated measure ANOVAs with accuracy and the inverse efficiency score as the dependent variables, including stimulation, phase, attention and conflict as within subject factors, which showed no significant main, or interaction effects.

**TABLE 3:** Results of the four-way ANOVA with stimulation, phase, conflict, and attention as within-subject factors for reaction time (RT) as dependent variable of the VSAD task.

Factor	df	F	*P*	η^2^
Stimulation	1	0.162	0.691	0.006
Phase	2	27.973	<0.001*	0.535
Conflict	1	12.618	0.001*	0.327
Attention	1	111.536	<0.001*	0.811
Stimulation × Phase	2	3.867	0.027*	0.129
Stimulation × Conflict	1	0.051	0.823	0.002
Stimulation × Attention	1	3.160	0.087	0.108
Stimulation × Phase × Conflict	2	0.550	0.580	0.021
Stimulation × Phase × Attention	2	2.958	0.061	0.102
Stimulation × Conflict × Attention	1	3.139	0.088	0.129
Stimulation × Phase × Conflict × Attention	2	1.672	0.198	0.060

df, degrees of freedom; η^2^, indicates generalized eta-squared. Significant results are marked with asterisks.

### 3.3 eSports performance results

**FIGURE 6:**
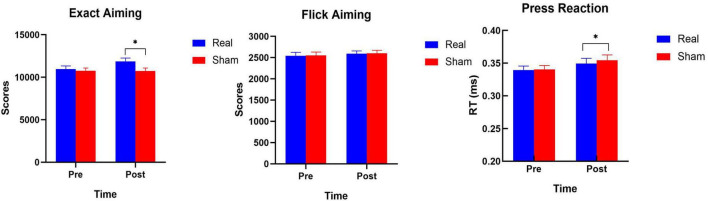
eSports skill performance before and after real, and sham stimulation. Significant results (*p* ≤ 0.05) of the *post-hoc t*-tests are marked with asterisks. The error bars show the standard error of means.

For the “Exact Aiming” scores, the main effect of stimulation [*F*_(1,26)_ = 4.738, *p* = 0.039, η^2^ = 0.154], but not of time was significant. The interaction between time and stimulation was, however, significant [*F*_(1,26)_ = 5.104, *p* = 0.032, η^2^ = 0.164] (Table 4). Follow-up *t*-tests showed that real and sham EA scores after, but not before intervention differed significantly (*p* = 0.004). The “Exact Aiming” scores after intervention were significantly larger in the real than in the sham stimulation group (11865 ± 395 vs. 10734 ± 345). For the “Flick Aiming” scores, the main effects of stimulation and time, as well as the respective interaction were not significant. With regard to “Press Reaction” reaction time, the ANOVA revealed a significant main effect of stimulation [*F*_(1,26)_ = 6.731, *p* = 0.015, η^2^ = 0.206]. However, the interaction between stimulation and time was not significant. Follow-up *t*-tests revealed a significant difference of “Press Reaction” scores between real and sham conditions only after intervention (0.340 ± 0.008 s vs. 0.354 ± 0.008 s, *p* = 0.036) (Table 5 and Figure 6). For “Exact Aiming” and “Flick Aiming,” larger scores are indicative for an improvement in gaming performance, suggesting enhanced gaming abilities. For “Press Reaction,” lower scores indicate a larger skill level.

**TABLE 4:** The two-way ANOVA with stimulation and time as within-subject factor of eSports performance as dependent variable.

Task	Factor	df	F	*P*	η^2^
Exact aiming	Stimulation	1	4.738	0.039*	0.154
	Time	1	4.006	0.056	0.133
	Stimulation × Time	1	5.104	0.032*	0.164
Flick aiming	Stimulation	1	0.025	0.876	0.001
	Time	1	1.817	0.189	0.065
	Stimulation × Time	1	0.003	0.958	<0.001
Press reaction	Stimulation	1	6.731	0.015*	0.206
	Time	1	0.325	0.573	0.012
	Stimulation × Time	1	0.209	0.652	0.008

df, indicates degrees of freedom; η^2^, indicates eta-squared. Significant results are marked with asterisks.

**TABLE 5:** The scores of eSports task performance (mean ± SE) in real and sham stimulation conditions pre- and post-intervention.

Items	Times	Scores (Mean ± SE)	
		Real	Sham
Exact aiming	Pre	10972.4 ± 352.595	10774.963 ± 301.905
	Post	11865.4 ± 395.391	10734.92 ± 345.004
Flick aiming	Pre	2544.467 ± 79.754	2550.074 ± 80.024
	Post	2590.974 ± 64.985	2600.593 ± 70.617
Press reaction	Pre	0.3396 ± 0.006	0.3404 ± 0.006
	Post*	0.3494 ± 0.008	0.3545 ± 0.008

SE, standard error of means. Significant results between real and sham groups are marked with asterisks.

## 4 Discussion

In this study we aimed to test the effect of 10 Hz tACS over the right frontal-parietal cortex on eSports skill performance. As expected, 10 Hz tACS improved eSports skill performance in healthy non-gamers. Compared to sham, the results of the real stimulation group showed a significant better performance in “Exact Aiming” and preliminary hints for improved performance in the “Press Reaction” task. Moreover, in the visuo-spatial distraction task, RT in the real, but not the sham stimulation group was significantly reduced, and thus performance was significantly improved during stimulation as compared to baseline in that group. This is the first study which investigated frontoparietal network effects of tACS on eSports skill performance, and the results suggest that neuromodulation via tACS might be feasible to improve eSports performance.

With respect to mechanistic reasons for the visual spatial attention distraction task performance improvement accomplished by tACS, van Schouwenburg et al. (2016) reported that HD-alpha tACS over the frontoparietal network enhanced top–down control over visual regions. In that study, compared to sham stimulation, synchronous frontoparietal alpha band stimulation of the right hemisphere enhanced alpha coherence between the frontal and parietal-occipital cortex (van Schouwenburg et al., 2016). The frontoparietal network is crucial for the control of attention, based on network communication through coherence in the alpha band (Noudoost et al., 2010; Zanto et al., 2011; Gilbert and Li, 2013; Heinen et al., 2014; Doesburg et al., 2016; Paneri and Gregoriou, 2017). However, the same group did not replicate these results in 2018, where they found no spatially selective effects of stimulation on behavior or coherence in in-phase and anti-phase stimulation protocols, compared to sham (van Schouwenburg et al., 2018). Reasons for this unexpected result may be that current density over F4 and P4 was threefold higher in the first study, and different spatial attention tasks were used in both studies. Indeed, (after-) effects of stimulation seem to increase with increasing current density (Nitsche and Paulus, 2000). In the present experiment, we used the small electrodes (3.14 cm^2^) applied in the study published in 2017, but stimulated for 20 min, and thus relevantly longer than that study (van Schouwenburg et al., 2016). The results of that study show that alpha frequency stimulation over the frontal and parietal cortices did improve performance, which was associated with a respective enhancement of fronto-parietal synchronization of alpha activity. This suggests that long-range alpha coherence is one mechanism by which the frontoparietal network controls spatial attention. The results of the present study suggest that this protocol improved visual attention ability with respect to RT during tACS, and this effect might be due to enhanced alpha band coherence between the frontal and parietal lobes. This is suggested by the significantly improved performance during stimulation in the real, but not the sham stimulation group relative to baseline. However, the respective between group difference at T1 was not significant, but showed only a trend, thus this study might have been underpowered to identify definite selective tACS effects in this task. At T2, and thus after stimulation, however, even this trendwise effect was absent, which might be in favor for a small, but specific effect of tACS during performance.

The visual spatial attention distraction task requires selective stimulus processing, including different variants of preparatory attention, such as spatial attention, feature-based attention, and object-based attention (Roelfsema et al., 1998; Treue and Martínez Trujillo, 1999; Buracas and Boynton, 2007; Snyder and Foxe, 2010; Noah et al., 2020), and thus mimics to a certain degree complex scene changes involved in action video games. Related studies have shown before that online alpha tACS improves attention, specifically it prevents attention decline (Clayton et al., 2019). This may be attributed to stronger attention alert in the visual field due to tACS of the right frontoparietal attention network (Posner and Petersen, 1990). Especially the alerting system has been associated with frontal and parietal regions of the right hemisphere, as has been shown for continuous performance and vigilance tasks (Fan et al., 2002; Petersen and Posner, 2012). Similar to the results of this study, a previous study reported that alpha tACS over the right dorsolateral prefrontal cortex improved performance of the arousal component of alertness and counteracted the typical vigilance decrement observed across time-on-task (Kemmerer et al., 2022). Therefore, tACS, as applied in the present study, might modulate visual attention via enhanced attention-related top-down control.

For eSports performance, the behavioral results showed that 10 Hz tACS over the right frontoparietal network improved performance significantly, as compared to the sham group, specifically in the “Exact Aiming” task. This task explores the ability to quickly capture many objects in a large game scene. A previous study demonstrated that alpha frequency tACS over the right frontal and parietal-occipital cortex increases long-range alpha coherence, which is one mechanism by which the frontoparietal network controls spatial attention (van Schouwenburg et al., 2016). The “Press Reaction” task requires conduction of a simple ballistic predefined movement. In the present study, press reaction time was less increased in the real as compared to the sham stimulation, and thus hints a performance improvement after real, as compared to sham stimulation. This result was, however, only evident for the *post-hoc* tests, but not the interaction of the respective ANOVA, is thus preliminary, and needs to be backed up by future studies. This result may be caused a stabilizing effect of tACS on visual attention (Clayton et al., 2019). For “Flick Aiming,” which was not improved in the present study, in contrast to the other tasks, fine motor control of the mouse is critical. In this task, the volunteer has to move the mouse continuously to control movement of an arrow to dots displayed on the screen, and then back to a stable circle. Therefore, in this task performance depends more on the ability to control the mouse exactly, and the motor component of that task is relatively dominant, as compared to the other tasks. The absence of a significant effect may thus be due to the fact that motion control depends mainly on the primary motor cortex, and the motor network (Krause et al., 2016; Schilberg et al., 2018), while we conducted stimulation over frontal and parietal cortices to enhance visual attention. Moreover, for motor control, β and γ frequencies are critical (Pogosyan et al., 2009; Miyaguchi et al., 2019). In the present study we focused, however, mainly on the attention component of gaming performance, and thus applied tACS in the α frequency band, which is relevant for improving fronto-parietal network coherence to enhance visuo-spatial attention (Lobier et al., 2018). In future approaches, it might also make sense to focus on the motor component, which is also relevant for gaming performance, and where tACS with different frequencies over the motor network has been shown to improve performance. Taken together, the results of the present study suggest that 10 Hz tACS of the right frontoparietal network improves some components of tasks related to of eSports skills in naïve participants. The application of this intervention might thus potentially improve sports performance. This was, however, not shown nor aimed for directly in the present study, and would require studies with repeated stimulation over prolonged time courses, and other intervention-optimizing stimulation approaches in the respective target population for the targeted activities. The results of such studies might have ethical implications, dependent on their outcomes. The World Anti-Doping Agency (WADA) has not classified electrical stimulation as a performance-enhancing substance at present and consequently this intervention does not face restrictions (Imperatori et al., 2018). However, neuro-doping is currently a vivid area under debate (Davis, 2013; Zhu et al., 2019), and while it is beyond the topic of this study to discuss this in detail, scientific information, such as delivered in the present study, is required as a foundation to come to scientifically informed regulatory decisions if this intervention should be rated as doping or not in future (Antal et al., 2022). Beyond application in sports, the positive cognitive effects of this intervention might make gaming combined with tACS a potentially attractive tool for rehabilitation in patients with cognitive deficits, which should be further explored. For potential application of this intervention in sports or clinical treatment, it should be taken, however, into account that this intervention has been most often been applied in a limited number of sessions, and with not optimized protocols, and respective studies would be required to explore its feasibility for application purposes in future.

Some limitations of this study should be taken into consideration. The design of this study was single blind, and principally double-blinding would have been advantageous. Since, however, the experimenter only communicated with the participants how to conduct the tasks before task performance, and the results of the blinding test show successful blinding, this limitation might have been minor in this specific case. Second, our sample consisted of a group of volunteers completely unfamiliar with eSports, and the eSports tasks were each conduced only once before and after intervention in each session. This might have resulted in suboptimal or floor effects. Enhanced effects might have been achieved with repetitive task performance after intervention via improved task learning caused by the intervention, and superior practice. Third, the transferability of these effects to skilled players cannot be taken for granted, but has to be explored in future studies, to explore the potential of the intervention for this group directly. Fourth, participants performed three consecutive visual attention task sessions, with less than 10 min between them, which might have led to cumulative effects independent from the intervention. In future studies, such practice effects should be avoided. Larger sample sizes in future studies would furthermore help to gain more clearly interpretable results of respective studies. The underlying physiology of the behavioral effects was not explored, thus mechanistic explanations remain speculative at present and future studies should add neuroimaging tools, such as EEG, and functional magnetic resonance tomography, to identify the physiological effects of this intervention. Furthermore, stimulation with the individual dominant alpha frequency has been suggested to be superior to stimulation with a standard frequency, and future studies should explore this option, and the return electrodes positioned between the target electrodes might have compromised efficacy of the stimulation to some degree because of antiphasic stimulation, thus alternative electrode positions should be probed.

## 5 Conclusion

The results of this study suggest that 10 Hz tACS over the right frontal and parietal cortex improves some aspects of eSports skill-related task performance in healthy students naïve to the tasks applied. HD-tACS improved visual spatial attention distraction task performance during stimulation, which might be due to enhanced alpha activity coherence between the frontal and parietal lobes. Since tACS also enhanced the ability to track multiple targets in a gaming task, we infer that this eSports performance improvement might have been caused by visual spatial attention enhancement. This is the first study which applied a tACS protocol to improve eSports performance, and the results supply preliminary hints that this intervention might be effective. However, this study was conducted in participants naïve to eSports gaming, and did not explore physiological mechanisms of these effects, which should be the topic of future studies. Moreover, the effects were relatively small, and likely short-lasting following this feasibility study. Therefore, we suggest that this tACS protocol might have principal potential as a neuromodulation tool to improve eSports athletes’ performance, however, the approach needs to be optimized to make it potentially applicable for eSports performance, but also for other applications, including rehabilitation training in patients with cognitive deficits.

## Data availability statement

The original contributions presented in the study are included in the article/Supplementary material, further inquiries can be directed to the corresponding author.

## Ethics statement

The studies involving humans were approved by the Ethical committee of Shanghai University of Sport (approval number: 102772022RT047). The studies were conducted in accordance with the local legislation and institutional requirements. The participants provided their written informed consent to participate in this study.

## Author contributions

FJ: Data curation, Formal Analysis, Writing – original draft. JZ: Data curation, Formal Analysis, Methodology, Writing – review and editing. MN: Supervision, Writing – review and editing. ZL: Data curation, Writing – original draft. YM: Formal Analysis, Writing – review and editing. YL: Funding acquisition, Supervision, Writing – review and editing.

## Footnotes

1
https://aim400kg.com/
