# Supplementary material for: Application of transcranial alternating current stimulation to improve eSports-related cognitive performance
Source: Front Neurosci. 2024 Feb 27;18:1308370. doi: 10.3389/fnins.2024.1308370 (PMC10927847; doi:10.3389/fnins.2024.1308370)
Supplement: Supplementary file 3 [file Table_3.DOCX]

DATASET ACTIVATE 数据集1.

GLM EA11 EA12 EA21 EA22

/WSFACTOR=Stimulation 2 Polynomial Time 2 Polynomial

/METHOD=SSTYPE(3)

/PLOT=PROFILE(Time*Stimulation)

/EMMEANS=TABLES(Stimulation) COMPARE ADJ(BONFERRONI)

/EMMEANS=TABLES(Time) COMPARE ADJ(BONFERRONI)

/EMMEANS=TABLES(Stimulation*Time)

/PRINT=DESCRIPTIVE ETASQ HOMOGENEITY

/CRITERIA=ALPHA(.05)

/WSDESIGN=Stimulation Time Stimulation*Time.

GLM FA11 FA12 FA21 FA22

/WSFACTOR=Stimulation 2 Polynomial Time 2 Polynomial

/METHOD=SSTYPE(3)

/PLOT=PROFILE(Time*Stimulation)

/EMMEANS=TABLES(Stimulation) COMPARE ADJ(BONFERRONI)

/EMMEANS=TABLES(Time) COMPARE ADJ(BONFERRONI)

/EMMEANS=TABLES(Stimulation*Time)

/PRINT=DESCRIPTIVE ETASQ HOMOGENEITY

/CRITERIA=ALPHA(.05)

/WSDESIGN=Stimulation Time Stimulation*Time.

GLM PR11 PR12 PR21 PR22

/WSFACTOR=Stimulation 2 Polynomial Time 2 Polynomial

/METHOD=SSTYPE(3)

/PLOT=PROFILE(Time*Stimulation)

/EMMEANS=TABLES(Stimulation) COMPARE ADJ(BONFERRONI)

/EMMEANS=TABLES(Time) COMPARE ADJ(BONFERRONI)

/EMMEANS=TABLES(Stimulation*Time)

/PRINT=DESCRIPTIVE ETASQ HOMOGENEITY

/CRITERIA=ALPHA(.05)

/WSDESIGN=Stimulation Time Stimulation*Time.

ATASET ACTIVATE 数据集1.

GLM RT1111 RT1112 RT1121 RT1122 RT1211 RT1212 RT1221 RT1222 RT1311 RT1312 RT1321 RT1322 RT2111

RT2112 RT2121 RT2122 RT2211 RT2212 RT2221 RT2222 RT2311 RT2312 RT2321 RT2322

/WSFACTOR=stimulation 2 Polynomial time 3 Polynomial conflict 2 Polynomial attention 2 Polynomial

/METHOD=SSTYPE(3)

/PLOT=PROFILE(time*stimulation)

/EMMEANS=TABLES(stimulation) COMPARE ADJ(BONFERRONI)

/EMMEANS=TABLES(time) COMPARE ADJ(BONFERRONI)

/EMMEANS=TABLES(conflict) COMPARE ADJ(BONFERRONI)

/EMMEANS=TABLES(attention) COMPARE ADJ(BONFERRONI)

/EMMEANS=TABLES(stimulation*time)

/EMMEANS=TABLES(stimulation*conflict)

/EMMEANS=TABLES(stimulation*attention)

/EMMEANS=TABLES(time*conflict)

/EMMEANS=TABLES(time*attention)

/EMMEANS=TABLES(conflict*attention)

/EMMEANS=TABLES(stimulation*time*conflict)

/EMMEANS=TABLES(stimulation*time*attention)

/EMMEANS=TABLES(stimulation*conflict*attention)

/EMMEANS=TABLES(time*conflict*attention)

/EMMEANS=TABLES(stimulation*time*conflict*attention)

/PRINT=DESCRIPTIVE ETASQ HOMOGENEITY

/CRITERIA=ALPHA(.05)

/WSDESIGN=stimulation time conflict attention stimulation*time stimulation*conflict time*conflict

stimulation*time*conflict stimulation*attention time*attention stimulation*time*attention

conflict*attention stimulation*conflict*attention time*conflict*attention

stimulation*time*conflict*attention.
